# Supplementary material for: Regulation of Transporters for Organic Cations by High Glucose
Source: Int J Mol Sci. 2023 Sep 13;24(18):14051. doi: 10.3390/ijms241814051 (PMC10531077; doi:10.3390/ijms241814051)
Supplement: Supplementary file 1 [file ijms-24-14051-s001.zip › ijms-2564129-supplementary.pdf]

Supplementary Table S1. List of primer sequences used for real-time PCR detection of SLC22A1 (hOCT1), SLC22A2 (hOCT2), and SLC47A1 (hMATE1), and glyceraldehyde-3-phosphatedehydrogenase (GAPDH).

Primer		Sequences (5′→ 3′)
OCT1	Forward	CAT CAT AAT CAT GTG TGT TGG CC
	Reverse	CAA ACA AAA TGA GGG GCA AGG CTT
OCT2	Forward	CGC CAT TCC TGG TCT ACC GGC
	Reverse	GCT TCC TCG ATG GTC TCA GGC
MATE1	Forward	AAG CTG GAG CTG GAT GCA GTC
	Reverse	CAG CAG AGG AGC AGG ACG AGC
GAPDH	Forward	CAA GCT CAT TTC CTG GTA TGA C
	Reverse	GTG TGG TGG GGG ACT GAG TGT GG

Supplementary Table S2. Ct values obtained by real-time PCR detection of SLC22A1 (hOCT1), SLC22A2 (hOCT2), and SLC47A1 (hMATE1), and glyceraldehyde-3-phosphatedehydrogenase (GAPDH) in HEK293 cells stably transfected with the respective transporter.

Cell line	mRNA	Ct value (mean ± SEM)
hOCT1-HEK293	hOCT1	22.4 ± 0.3, N = 4
	GAPDH	15.3 ± 0.2, N = 4
hOCT2-HEK293	hOCT2	16.2 ± 0.3, N = 4
	GAPDH	14.2 ± 0.7, N = 4
hOCT3-HEK293	hOCT3	22.1 ± 1.2, N = 4
	GAPDH	15.6 ± 0.2, N = 4
hMATE1-HEK293	hMATE1	17.9 ± 0.1, N = 4
	GAPDH	15.9 ± 0.1, N = 4

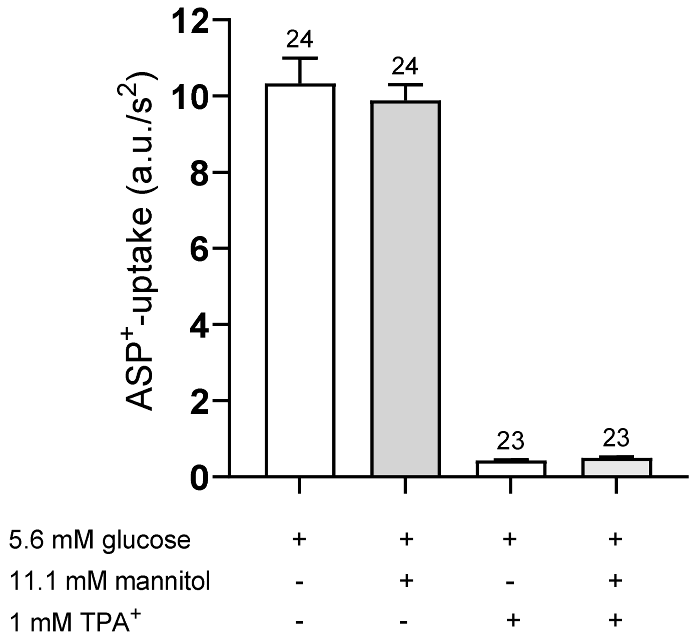

Supplementary Figure S1. Effect of increased medium osmolarity by addition of 11.1 mM mannitol on ASP+ uptake rates (determined in the presence or not of 100 nM TPA+) in hOCT2-HEK293 cells after 48 h incubation. Values are means ± SEM of initial fluorescence increase in arbitrary units (a.u.)/s² calculated from 23–24 replicates measured in at least 3 independent experiments. The number of replicates is reported on every column. The addition of mannitol did not change ASP+ uptake rates.

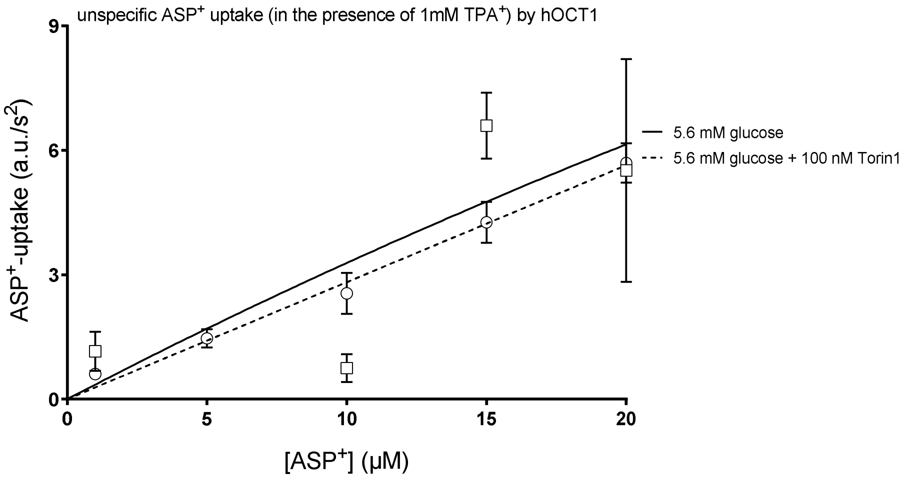

Supplementary Figure S2. Determination of ASP+ unspecific uptake rates (determined in the presence of 100 nM TPA+) in hOCT1-HEK293 cells after 48 h incubation with 5.6 mM glucose in the presence (open squares) or not (open circles) of 100 nM Torin 1. Values are means ± SEM of initial fluorescence increase in arbitrary units (a.u.)/s² calculated from 3-23 replicates measured in at least 3 independent experiments.

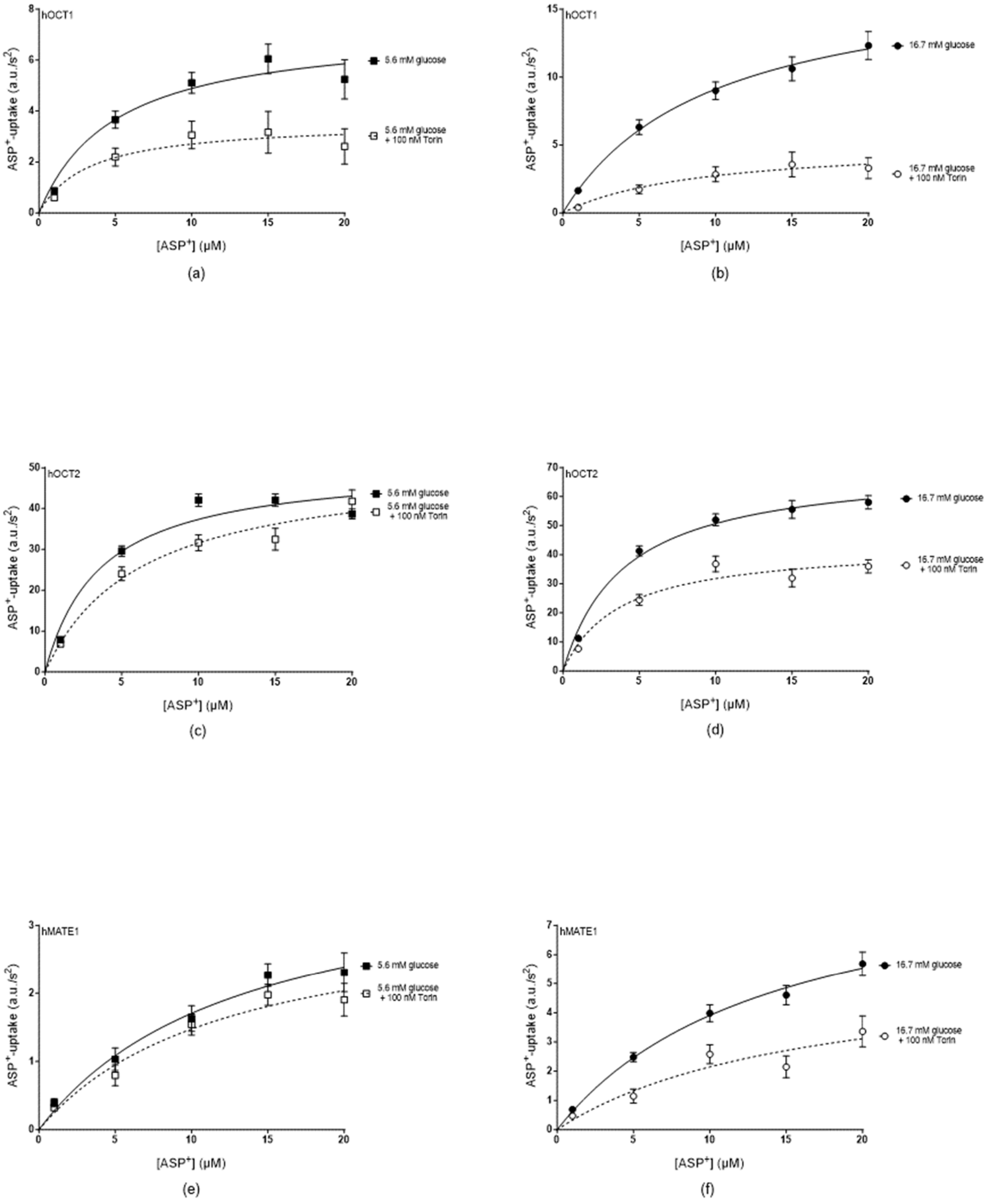

Supplementary Figure S3. ASP^+ specific uptake rates in hOCT1- (panels **a and b**), hOCT2- (panels **c and d**) and hMATE1- (panels **e and f**) HEK293 cells after 48 h incubation with 5.6 (closed squares, panels **a,c, and e**) or 16.7 mM glucose (closed circles, panels **b, d, and f**) compared to that measured in the presence of 100 nM Torin-1 (open squares for measurements under 5.6 mM glucose in panels **a,c, and e** and open circles for measurements under 16.7 mM glucose in panels **b, d, and f**). Values are mean ± SEM of initial fluorescence increase in arbitrary units (a.u.)/s^2 calculated from 19–63 replicates/concentration measured in at least 3 independent experiments.

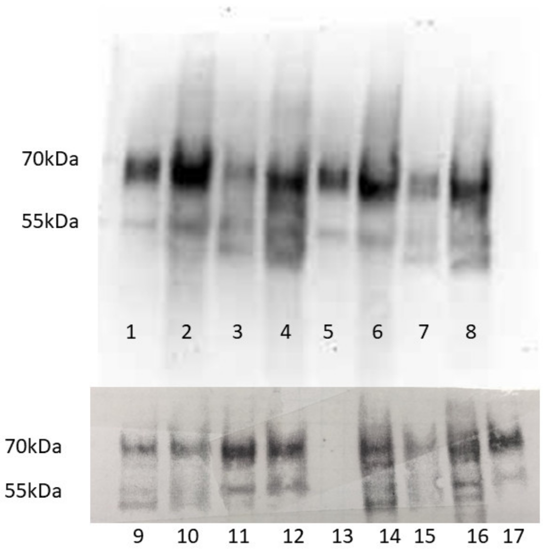

1 Biotinylated lysate 1 after incubation with 16.7 mM glucose
2 Total lysate 1 after incubation with 16.7 mM glucose
3 Biotinylated lysate 2 after incubation with 5.6 mM glucose
4 Total lysate 2 after incubation with 5.6 mM glucose
5 Biotinylated lysate 3 after incubation with 16.7 mM glucose
6 Total lysate 3 after incubation with 16.7 mM glucose
7 Biotinylated lysate 4 after incubation with 5.6 mM glucose
8 Total lysate 4 after incubation with 5.6 mM glucose
9 Biotinylated lysate 5 after incubation with 5.6 mM glucose
10 Total lysate 5 after incubation with 5.6 mM glucose
11 Biotinylated lysate 6 after incubation with 16.7 mM glucose
12 Total lysate 6 after incubation with 16.7 mM glucose
13 Empty lane
14 Biotinylated lysate 7 after incubation with 5.6 mM glucose
15 Total lysate 7 after incubation with 5.6 mM glucose
16 Biotinylated lysate 8 after incubation with 16.7 mM glucose
17 Total lysate 8 after incubation with 16.7 mM glucose

Supplementary Figure S4. Investigation of hOCT2 expression in the plasma membrane by biotinylation experiments. The panels show Western blot analysis of hOCT2 expression in biotinylated fraction and total cell lysates from cell incubated for 48 h in the presence of 5.6- or 16.7-mM glucose. The contents of the lanes are explained in the legend on the right. The lane 13 is empty. Molecular dimensions in kDa are indicated by the numbers on the left.
